# The Herpes Zoster Patient Pathway and Gaps in Current Vaccination Guidelines in Southeast Asia: Summary of a Zoster Experts’ Network Scientific Workshop

**DOI:** 10.3390/vaccines12121433

**Published:** 2024-12-19

**Authors:** Gyneth Lourdes G. Bibera, Peter San Martin, Desiree A. M. van Oorschot, Afif Nurul Hidayati, Deliana Permatasari, Sasheela Sri La Sri Ponnampalavanar, Kughan Govinden, Maria Christina Filomena Batac, Joselito Javier, Terapong Tantawichien, Phatu Boonmahittisut, Trinh Minh Trang, Thanh Tuyen Dang Thi

**Affiliations:** 1 GSK, Taguig City 1634, Philippines; 2 GSK, 1300 Wavre, Belgium; 3 Department of Dermatology, Venereology and Aesthetics, Faculty of Medicine, Universitas Airlangga, Surabaya 60115, Indonesia; 4 GSK, Jakarta 12930, Indonesia; 5 Department of Medicine, University of Malaya, Kuala Lumpur 50603, Malaysia; 6 GSK, Petaling Jaya 47800, Malaysia; 7 Department of Dermatology, University of the Philippines—Philippine General Hospital, Manila 1000, Philippines; 8 Division of Infectious Diseases, Department of Medicine and Tropical Medicine, Chulalongkorn University, Bangkok 10330, Thailand; 9 GSK, Bangkok 10330, Thailand; 10 National Hospital of Dermatology and Venereology, Hanoi City 10000, Vietnam; 11 GSK, Ho Chi Minh City 70000, Vietnam

**Keywords:** Southeast Asia, herpes zoster, shingles, vaccination, recombinant zoster vaccine, zoster vaccine live, patient pathway

## Abstract

The burden of herpes zoster (HZ) is recognized worldwide; however, there is seemingly limited information on incidence and vaccination practices in Southeast Asia (SEA). A scientific workshop was held by the Zoster Experts’ Network to exchange and consolidate insights on the burden of HZ and the patient pathway in SEA. The workshop included practicing clinical experts and public health specialists/epidemiologists from Indonesia, Malaysia, the Philippines, Thailand, and Vietnam. It aimed to identify gaps in the literature, outline patient pathways, and evaluate HZ vaccine recommendations among these countries. Consensus was identified on the substantial lack of epidemiological data on HZ in SEA and the need to investigate the impact of age, immunocompromising conditions, and comorbidities on the incidence and severity of HZ in the region. However, available data in SEA did indicate a rising disease and socioeconomic burden of HZ, with concerns that current treatment strategies for HZ are suboptimal. The HZ patient pathways generated by the experts highlighted common themes and differences between the five countries. Furthermore, the experts highlighted the lack of awareness of HZ and its impact on patients’ quality of life, among patients and healthcare professionals. Evaluation of the current local HZ vaccine recommendations further showed differences in age and the inclusion of at-risk populations between countries. The workshop outcomes emphasize the need for further HZ surveillance in SEA. Efforts to align and address leakage within the patient pathway and raise awareness on the impact of HZ should be prioritized. Awareness initiatives and alignment on vaccine recommendations are also needed.

## 1. Introduction

Herpes zoster (HZ), otherwise known as shingles, is a blistering dermatomal rash that can cause long-term intense pain, among other complications, and can impact patients’ quality of life [1,2]. HZ is caused by the reactivation of latent varicella zoster virus (VZV) in sensory ganglia [2,3]. Following primary infection, VZV becomes dormant and asymptomatic in the nerval tissue, but may become reactivated if the cell-mediated immunity controlling viral replication is lost [3]. Such reactivation causes VZV viral particles to enter dermatomes, leading to inflammation and development of HZ. The lifetime risk of developing HZ, without vaccination, ranges between 20% and 30% [4,5].

The age-standardized incidence of VZV is estimated to be 1198.7 per 100,000 individuals worldwide, with a slight upward trend seen in recent years [6]. In Southeast Asia, previous reports of VZV seroprevalence vary, with seropositivity generally increasing with age, ranging from 10.2% in Thailand (infants aged <1 year) to 96.4% in Singapore (adults aged 70–79 years) [7]. For HZ, global cumulative incidence is estimated at 2.9–19.5 cases per 1000 individuals, with reports showing trends of increasing incidence over time across North America, Europe, and the Asia-Pacific region [8]. However, recent systematic literature reviews have identified the Asia-Pacific region, and notably Southeast Asia, as significantly underrepresented in HZ epidemiological studies, despite a clear presence of disease being reported from single centers in the region [7,9].

HZ can lead to various disease complications, including postherpetic neuralgia (PHN), cutaneous (e.g., disseminated zoster and bacterial superinfections), ocular (HZ ophthalmicus), and neurologic (e.g., encephalitis and meningitis) complications, leading to significant patient burden [10,11,12]. These complications lead to significant hospitalization rates, with previous global estimates of HZ-associated hospitalization of up to 25/100,000 person-years [13]. Estimates have indicated that at least 50% of patients hospitalized with HZ experience complications of PHN, secondary infections, or inflammatory sequelae [9]. Although hospitalization data are scarce in Southeast Asia, one study in Singapore reported 3% of hospitalizations to be related to HZ, which was substantially higher than that reported in other regions, including Europe [7]. Individuals with immunocompromising conditions and older adults are particularly vulnerable to HZ infection and severe clinical outcomes that can lead to increased hospitalization rates [3,13,14,15,16].

Additionally, insights from global data also support the idea that co-morbidities, including diabetes mellitus, human immunodeficiency virus (HIV), and chronic obstructive pulmonary disorder, increase the risk of developing HZ and associated complications [17,18,19]. Given that the Southeast Asian aging population is rapidly expanding, with expectations that it will more than double in 2050 (20.3%) from 2017 (9.8%) [20], the burden of HZ is expected to further increase; hence, there is significant need for preventative options for HZ. Currently, two types of vaccines are available for the prevention of HZ, namely the recombinant zoster vaccine (RZV) and zoster vaccine live (ZVL), which differ in the presence of live VZV [7,21]. Despite the availability of these vaccines in many countries, including those in Southeast Asia, few have implemented HZ vaccination into national immunization programs.

A new initiative labelled the Zoster Experts’ Network (ZEN), involving clinical experts and public health specialists from five Southeast Asian countries (Indonesia, Malaysia, the Philippines, Thailand, and Vietnam), has been established with the aim of evaluating and addressing the unmet needs of patients with HZ in the region. Here, we present the outcomes of the first ZEN scientific workshop, held in 2023, which focused on evaluating the patient pathway, gaps in existing vaccination guidelines, and clinical considerations for vaccination at a country level in Southeast Asia for patients with HZ. Experts discussed the current landscape of HZ in their respective countries, calling from their clinical experience and the literature. The outcomes and consensus of the workshop are summarized in this article.

## 2. Methods

The ZEN is a learning forum established to provide an avenue for scientific exchange on HZ disease burden and prevention in Southeast Asia. It includes practicing clinical experts and public health specialists/epidemiologists residing in five Southeast Asian countries: Indonesia, Malaysia, the Philippines, Thailand, and Vietnam.

A scientific workshop was held to bring together the ZEN participants, commemorating the first activity of the ZEN initiative. The objectives of the workshop were to build a representative and common understanding of the burden and impact of HZ disease in Southeast Asia at a country level, identifying gaps in the literature as well as in current recommendations for HZ vaccines. A key objective was also to outline the current patient pathway across the experts’ respective countries and define a consensus of activities that will benefit patients and reduce the burden of HZ.

The workshop was structured by two main activities:

1. Regional experts in HZ presented the latest epidemiological data on HZ, focusing on the health and socioeconomic burden of HZ globally and in Southeast Asia, followed by open discussions with the ZEN forum to outline key insights and data gaps.
2. An interactive session to outline and discuss the patient pathway for patients with HZ, current adult immunization landscape, and guideline recommendations for HZ in each expert’s respective country. Each country presented their outputs, with discussion among the forum.

The consensus and outcomes of the workshop were summarized in a collaborative executive summary. This manuscript presents an overview of the data gaps on the burden of HZ in Southeast Asia, the patient pathway for HZ patients, and practices for adult immunization for HZ at a country level, as informed by the expert consensus in the ZEN scientific workshop.

## 3. Results

On 26 August, 2023, the first scientific workshop of the ZEN Forum was held in Bangkok, Thailand. A total of 24 expert clinicians, public health specialists, and epidemiologists from five Southeast Asian countries (Indonesia, Malaysia, the Philippines, Thailand, and Vietnam) participated in the workshop, spanning several clinical specialties including dermatology, geriatrics, infectious diseases, immunology, neurology, and rheumatology (Table S1).

The key discussion points of the workshop were current insights on HZ epidemiology in Southeast Asia, including HZ complications, the socioeconomic burden of HZ, and current treatment pathways, and preventive measures for HZ in Southeast Asia. The results of these discussion points are described below.

### 3.1. Current Insights on HZ Epidemiology in Southeast Asia

Experts at the ZEN forum agreed that there is a lack of data on HZ epidemiology in Southeast Asia, despite reports of increasing incidence and disease burden of HZ globally.

A systematic literature review, published in 2017, reported an average HZ incidence of 3–10 per 1000 person-years in the Asia-Pacific, rising by approximately 5% per year [9], similar to global figures [8]. However, these data are not representative of Southeast Asia, given that data were only available from Thailand. This scarcity of regional data was also evident in a systematic literature review published in 2023, which summarized the latest data on the epidemiologic and clinical characteristics of HZ in six Southeast Asian countries [7]. Studies reporting country-wide estimates of HZ incidence in Southeast Asia were only available from the Thailand Bureau of Epidemiology, which reported 0.26 cases of HZ per 1000 person-years in 2008 [22].

Prevalence data in Southeast Asia are available; however, they are primarily derived from single-center studies in the outpatient setting [7]. In Thailand, country-level estimates for the number of HZ cases were 6.44 and 7.65 cases per 100,000 individuals in 2001 and 2014, respectively [9]. Prevalence estimates were also available from the Philippines and Indonesia, and ranged between 0.68–0.7%, with most patients aged ≥45 years; however, these were based on single-center studies [23,24]. Data were also presented at the workshop from the National Hospital of Dermatology and Venereology (NHDV) in Vietnam, which indicated a prevalence of HZ at 0.9% in 2022, with similar reports in quarter (Q)1–Q2 of 2023 (Table S2). Consistent with other epidemiological reports, the majority of patients were female and aged >50 years [7,9].

In conclusion, data are limited on HZ epidemiology in Southeast Asia, which is likely a result of a lack of surveillance and scarcity of research in the region, alongside a potential lack of awareness among patients and healthcare professionals [7]. Hence, the consensus from the ZEN participants is that there is a strong need for comprehensive surveillance on HZ in Southeast Asia, which should be considered a priority.

#### 3.1.1. HZ Complications and Effect of Comorbidities

At the ZEN Forum, participants recognized the importance of the various disease complications associated with HZ.

Older adults and people with immunocompromising conditions are at a higher risk of developing HZ complications versus younger or immunocompetent populations [10,11,12]. For example, a retrospective analysis of patients diagnosed with PHN in Malaysia revealed a predominant occurrence among adults aged 61–70 years, with approximately 26.0% of these patients also having immunocompromising conditions such as diabetes and malignancy [25]. A global systematic review of data from 26 countries reported an estimated hospitalization rate associated with HZ infection of 2–25 per 100,000 person-years, with higher rates among older adults [13].

In Southeast Asia, although reported from single-center or case studies, the impact of HZ-related complications is evident; 10.2–85% of HZ cases are reported to have at least one complication, with PHN having been identified in 6.3–50% of patient populations examined [7]. HZ involvement of the first branch of the trigeminal nerve (HZ ophthalmicus) was also commonly reported among patients in Southeast Asia (4.98–28.57%) [7], with a similar rate of HZ ophthalmicus observed in a longitudinal study of 633,474 cases of HZ in the United States (US; 7.9%) [26]. A concerning observation at the NHDV was also an increased rate of hospitalization associated with HZ in Vietnam in 2023 compared with 2022 (Table S2), which highlights the need for further evaluation on the burden of HZ complications on healthcare systems in the region.

Guidelines in countries such as the US recommend HZ vaccination in individuals who are immunosuppressed because of underlying conditions or related therapy [27]. However, there are limited published data on the risk of infection among patients with immunocompromising conditions in Southeast Asia. In particular, HIV is of concern, given that HIV is a continuing challenge in countries such as the Philippines [28], and the incidence of HZ is associated with HIV [29]. Considering that an estimated 29% of HIV cases in the Philippines are identified at an advanced disease state [28], the impact of immunocompromised status on the incidence and severity of HZ should be a focus in future efforts to collect epidemiological data.

Diabetes has also been shown to be a significant risk factor for HZ [30]; this is a concern for Southeast Asia, where over 90 million patients with diabetes aged 20–79 were identified in 2021 by the International Diabetes Federation, with the highest prevalence reported in Malaysia (20.0%) [31]. However, limited data exist on the relationship between diabetes and HZ. One study evaluating a 10-year cohort of Thai patients with diabetes mellitus (N = 1428) reported an incidence rate for HZ of 3.96 per 1000 person-years and identified other independent risk factors for HZ including hypertension, hypoglycemic therapy, and herbal remedy use [32]. However, these data were derived from a single center, as with most other studies on HZ in the region [7].

Furthermore, the increasing aging population in the region highlights the growing need to evaluate populations at risk of developing HZ [20]. This is pertinent considering that a trend of increasing incidence of HZ with increasing age is evident worldwide [8].

#### 3.1.2. Socioeconomic Burden of HZ

Although limited, available data in Southeast Asia indicate a similar and rising socioeconomic burden of HZ as reported globally [9,33]. Most data originate from Thailand, with one study reporting the estimated mean direct healthcare cost for HZ at 3083.39 ± 5047.03 Thai baht (THB; approximately 90.41 ± 148.0 US dollars [USD]) between 2007–2008. This same study also found social factors to be affected by HZ, including a median loss of 5 days of work (1–8 days) among family members acting as caregivers [22].

### 3.2. Current Treatment and Preventive Measures for HZ in Southeast Asia

Another concern highlighted by the ZEN forum was that current treatment strategies for HZ are suboptimal. The US’ Advisory Committee on Immunization Practices (ACIP) recommends that antiviral treatment is initiated within 72 h of acute HZ; however, this timeframe is typically difficult to achieve in real-world practice [14]. HZ-related complications are also difficult to treat, with no curative measures available for complications such as PHN [34]. Pain arising from HZ infection is managed through palliative treatments, which can involve multiple lines of medications, including, but not limited to, gabapentinoids, corticosteroids, tricyclic antidepressants, and opioids [34].

In Malaysia, one retrospective, single-center study showed that over half of patients (54.8%; N = 179) were late in seeking treatment, with a median disease duration of 4 days before intervention [35]. Based on discussions at the ZEN workshop, knowledge gaps among healthcare professionals in diagnosing HZ were highlighted, including difficulty in recognizing atypical presentations of HZ and in differentiating from other similarly presenting infections such as herpes simplex, folliculitis, and impetigo. The experts also noted inconsistency in dosage of antivirals prescribed for HZ.

#### 3.2.1. Patient Pathways for HZ

In the ZEN scientific workshop, the participants collectively outlined the current patient pathway from experience for individuals presenting with symptoms of HZ in their respective countries (Table 1; Figures S1–S5). Each pathway generally involved initial medical consultation with either a general practitioner or dermatologist, which may be followed by treatment with antivirals or referral to other specialists. Diagnostic testing for HZ occurred late in the curated patient pathways, and was not highlighted in those produced by the Malaysian (Figure S2) and Vietnamese (Figure S5) clinicians during the workshop. The pathways outlined for Indonesia (Table 1; Figure S1), Malaysia (Table 1; Figure S2), the Philippines (Table 1; Figure S3), and Vietnam (Table 1; Figure S5) indicated that some patients initially seek alternative medical care or self-treatment, before seeking medical consultation, which can result in late presentation to healthcare facilities and delays in receiving definitive treatment.

Experts from Thailand highlighted a lack of clarity on when and where to seek adequate medical care for symptoms of HZ (Figure S4), which may apply generally to other countries in the region. Patients are often dependent on clinical presentation and appearance of a rash to gain a diagnosis, and since pain can often precede rash onset, misdiagnoses are common [36]. These factors can delay pertinent timing for treatment with antivirals [14,36]. The mean duration of symptoms before consultation has been reported as 4 days (range: 1–21 days) in Thailand and 8 ± 2.1 days in the Philippines [7], consistent with the opinions of the ZEN on suboptimal timing of treatment. Another difference observed between pathways was an inconsistency in the level of care supported by governmental bodies and/or universal coverage. This could be a factor leading to underestimation of HZ incidence and associated costs in the region. Therefore, consensus on streamlining the patient pathway at a country and regional level was identified as critical to ensure early diagnosis and, in turn, adequate treatment. Misdiagnosis of HZ was also raised in the forum, leading to suggested efforts for society-led online education programs as an effective strategy to combat these issues and increase awareness of HZ.

#### 3.2.2. Insights on Recommendations for HZ Vaccination

As current treatment strategies for HZ may be suboptimal or difficult to adequately implement in real-world practice, preventative strategies against HZ are pertinent.

In phase III clinical trials (ZOE-50 and ZOE-70), RZV has demonstrated >90% vaccine efficacy against HZ after seven years post-vaccination in adults aged ≥50 or ≥70 years [37]. Long-term follow up of vaccinated patients has also demonstrated strong and consistent RZV efficacy, reported at 89.0% after a mean of 9.6 years post-vaccination [38]. A two-dose schedule for RZV is also recommended by ACIP for all adults aged ≥50 years [39]. A clinical trial of ZVL has also demonstrated vaccine efficacy of 61% in adults aged ≥60 years over a median surveillance period of 3.1 years (range: 1.0–4.9 years) [40].

In addition to adults aged ≥50 years, RZV has also demonstrated efficacy in immunocompromised adults aged ≥18 years, including in those with HIV, hematological malignancies, and organ transplant recipients [41]. For example, vaccine efficacy was reported to be 68% and 87% in autologous hematopoietic stem cell transplant recipients and adults with hematological malignancies, respectively [41,42,43]. Based on such efficacy data, ACIP also recommends two doses of RZV for adults aged ≥19 years who are immunodeficient or immunosuppressed [27].

Current guidelines for HZ vaccination in Indonesia, Malaysia, the Philippines, Thailand, and Vietnam are summarized in Table 2. Despite the availability of vaccines against HZ, many Asian countries have not implemented HZ vaccination into widespread clinical practice, the causes of which need to be addressed. Additionally, although the guidelines summarized in Table 2 suggest that physicians recommend HZ vaccination to certain patients, at the time of publication, none of the countries included offer HZ vaccination as part of their national vaccination systems free of charge, further limiting uptake. The majority of guidelines included vaccination for older adults and/or those with immunocompromising conditions due to a higher risk of severe outcomes from HZ infection [7,15,44]. However, this workshop highlighted differences in current guidelines on HZ vaccination in Southeast Asia. For example, variations are present with respect to the precise cut-off on patients’ age (≥50 years or ≥60 years) across recommendations in the region and on whether a prior history of HZ or VZV infection should be considered. Critically, Indonesia do not currently recommend vaccination for immunocompromised patients [45]. despite vaccination among these populations being recommended globally in countries such as in the US and United Kingdom [27,46].

#### 3.2.3. Challenges in Implementing HZ Vaccination in Practice

In the ZEN scientific workshop, participants were requested to outline and discuss the adult immunization pathway for HZ in their countries. There was consensus on the lack of awareness among patients on the importance of vaccination as a preventative measure against HZ, with clinicians in Vietnam reflecting that current practices usually prioritize chronic disease management over vaccination. A lack of awareness among healthcare providers was also raised, highlighting the importance of medical education programs curated for HZ vaccination, vaccine efficacy, safety, and local HZ disease burden. Gaps in knowledge and awareness of HZ vaccines has also been reported in other regions, including Turkey and the US [55,56]; hence, awareness campaigns on the availability of vaccines should also be considered in Southeast Asia.

Additionally, social media was highlighted as a key contributor to healthcare awareness in all residing countries. Consideration should be put towards social media campaigns and on ensuring influencers, patients, and communities are engaged to raise awareness of HZ infection and vaccination. However, caution should be taken, with lessons from the coronavirus disease 2019 (COVID-19) pandemic, in which disinformation campaigns formed on social media platforms, which could promote vaccine hesitancy and reduce coverage [57].

The lack of vaccine recommendations integrated into patient pathways highlighted in this workshop (Figures S1–S5), particularly during discharge, may also reflect a lack of awareness of HZ vaccination in current practices or across specialties. Effective collaboration is also needed among healthcare specialties, considering that patients with HZ can have a range of symptoms at presentation, which can lead to seeking medical consultation from clinicians across multiple different specialties.

Another major concern raised as a deterrent to vaccination was the affordability of vaccines. Strategies should be implemented to lower vaccine costs and make them more accessible to a wider range of patients. Exploring payment instalment options and collaborating with insurance companies to cover preventive measures may circumvent this potential issue in Southeast Asia. Advocations for a revolving fund in Southeast Asia for vaccines have also been published in recent years, particularly in the context of the COVID-19 pandemic [58].

A summary of the key barriers to HZ vaccination highlighted in the workshop is presented in Table 3. The consensus of the experts was that the key barriers were a lack of awareness among patients and healthcare workers on the burden of HZ and benefits of HZ vaccination and socioeconomic restrictions, limiting the ability for some patients to be vaccinated against HZ. In response to these barriers, key consensus actions were suggested by the experts to aid understanding of and reduce the burden of HZ in Southeast Asia; these are summarized in Table 4. Suggested actions included driving data collection related to HZ, alongside efforts to improve curation and distribution of medical education programs for healthcare professionals on HZ burden and vaccination guidelines. Furthermore, improving public awareness on HZ vaccination, addressing issues of vaccine availability and affordability, and standardizing current practices in the HZ patient pathway provide additional methods for reducing HZ burden in the region (Table 4).

## 4. Discussion

The outcomes of this ZEN scientific workshop further highlight the limited data on HZ in Southeast Asian countries. Although only five countries from the region were represented, these countries collectively amount to a large proportion of the regional population and are among those previously highlighted to have sparse insight on HZ disease burden and HZ vaccine safety and efficacy [7,9].

An advantage of the ongoing ZEN initiative is the variety of expertise held by the participants, covering a wide spectrum of clinical specialties. Perspectives from such experts at the ZEN will be critical to unify optimal patient care for HZ in the region. In particular, there is a strong consensus on the lack of epidemiological information and the impact of comorbidities placing populations at risk in the region. For example, multiple literature reviews of HZ epidemiology have highlighted the scarcity of data in Southeast Asia [7,9], with a 2023 review of HZ disease burden in Southeast Asia finding only two articles to report HZ incidence in the region, both of which were for Thailand [7].

The experts indicated a substantial lack of patient and healthcare professional awareness of HZ vaccination (Table 3). This lack of knowledge of HZ aligns with previous studies in Southeast Asia, including a 2016 survey of patient perception of HZ, which found over half of the 118 respondents to have inaccurate knowledge about the disease [59]. Poor HZ knowledge is also recognized in other countries worldwide, including those residing in Latin America, the Gulf Cooperation Council, and Turkey [55,60,61].

This workshop also highlighted the diverse patient pathways present in Southeast Asia, including variation in the healthcare professional that patients typically present to and in the pathways for uncomplicated and complicated HZ cases. Similar conclusions have been made in other regions, including in a 2022 review of HZ management in China [62]. Additionally, multiple steps in these patient pathways are not supported by governmental bodies and/or universal coverage, potentially leading to inaccuracies in estimating the cost of HZ in these regions. Although vaccines to protect against HZ are available, guidelines from the respective countries showed clear inconsistency in terms of recommendations based on age, weak versus healthy immune systems, and history of HZ or VZV infection. Another barrier to immunization is that HZ vaccines are not included in national immunization programs free of charge.

Based on the findings of the ZEN workshop, several recommendations were made to alleviate the burden of HZ in Southeast Asia. To address the scarcity of data on HZ epidemiology in the region, the ZEN recommends data collection initiatives on HZ presentation, epidemiology, and outcomes, which may be achieved by leveraging existing data available in other regions to raise awareness of the burden of HZ. Improving patient and healthcare professional knowledge of HZ and HZ vaccination may be achieved through targeted medical education programs and public awareness initiatives, while variation in the HZ patient pathway may be addressed by the development of guidelines to standardize current practices. Finally, issues regarding vaccine availability and affordability in Southeast Asia must be addressed to improve vaccine uptake in the region, which may be achieved by exploring payment installment options, collaborating with insurance companies to cover costs, or by investigating the feasibility of including HZ vaccination into immunization programs.

The ZEN workshop focused on establishing a common understanding of the impact HZ and the patient pathway in Southeast Asia. Although further discussion is required to develop implementation strategies for the recommendations made, next steps may include the formulation of rollout plans for public awareness initiatives or analyses of the budget impact of implementing HZ vaccination into national immunization programs.

## 5. Conclusions

This scientific workshop represents the first activity of an initiative led by the ZEN, which aims to improve patient care and outcomes related to HZ infection in Southeast Asia. Key consensus actions derived from the workshop include driving data collection on HZ, supporting the development of medical education programs on HZ, raising public awareness and improving accessibility of HZ vaccination, and standardizing current practices in the HZ patient pathway (Table 4). These steps will be critical to ultimately reducing the impact of HZ in Southeast Asia.

The formation of the ZEN provided a much-needed opportunity to share experiences between clinical experts and public health specialists, with further scope for collaboration between countries through research or development of public health initiatives to ultimately improve clinical and preventive care for HZ. The ZEN may also serve as inspiration for other regions that may benefit from an efficient method for engaging clinical experts to discuss targeted approaches for addressing the burden of HZ.

## Figures and Tables

**Table 1:** Pathway for patients with HZ in Southeast Asian countries.

	Indonesia	Malaysia	Philippines	Thailand	Vietnam
Do some patients delay seeking medical consultations?	Yes, may seek alternative/traditional treatment	Yes, may seek alternative/traditional treatment	Yes, may self-treat	Not discussed	Yes, may self-treat
First medical consultation for most cases	GP	GP/Family Medicine, Emergency Department, or another specialist (e.g., dermatologist, geriatrician, ID physician)	GP/Family Medicine, Internal Medicine/Geriatrics or dermatologists	GP	Dermatologist
First medical consultation for other cases	Complicated cases: Specialist (e.g., ENT, ophthalmologist, neurologist)		Severe pain: Emergency Department	Patients may consult a pharmacist or be reviewed by a specialist (e.g., endocrinologist, rheumatologist, oncologist)	Patients may consult a pharmacist or be seen by another specialist
	Immunocompromised patients: Internal Medicine		Dermatologist may perform/request supporting tests		
Defined pathways for uncomplicated and complicated HZ cases?	Yes				
Presence of government or healthcare insurance with regard to consultation and/or treatment?	Yes				
Vaccine education/recommendation provided?	Yes, vaccination recommended, and follow-up patient education provided following the resolution of symptoms	Yes, follow-up patient education provided following the resolution of symptoms	Yes, follow-up patient education provided following the resolution of symptoms	Yes, vaccination recommended (by <20% of HCPs ^1^)	Not discussed

^1^ Data reported from informal interviews with five Thai clinicians. ENT: ears, nose, and throat; GP: general practitioner; HCP: healthcare professional; HZ: herpes zoster; ID: infectious disease.

**Table 2:** Recommendations for HZ vaccination in Southeast Asian countries.

Country	Recommendations for Older Patients	Recommendations for Immunocompromised Patients
Indonesia	ZVL: ≥50 years, with or without a history of HZ infection [45] ^1^	No published recommendations
Malaysia	RZV: ≥50 years, with or without a history of varicella or HZ infection [47] ZVL: ≥60 years, with history of HZ infection, with or without a history of varicella infection [48,49] ^2^	RZV: ≥18 years and immunosuppressed [47] RZV and ZVL: Potential kidney transplant recipients aged ≥50 years [50]
Philippines	RZV: ≥50 years, with or without prior receipt of varicella vaccine or ZVL [51] ZVL: ≥60 years, without a history of HZ [51] ^3^ RZV and ZVL: ≥60 years, with apparently good health [52] ^4^	No published recommendations
Thailand	RZV: Aged >50 years, with or without a comorbidity (optional) [53] ZVL: >60 years, without immunocompromising conditions (optional) [53]	RZV: >18 years with immunocompromising conditions [53] ^5^
Vietnam	RZV and ZVL: ≥50 years [54] ^4,6^	RZV: ≥19 years with immunocompromising conditions and/or on immunosuppressive therapies [54] ^4^

^1^ ZVL contraindicated in pregnant individuals and patients with HIV (not receiving ART) or immunocompromising conditions; ^2^ ZVL contraindicated in individuals aged >80 years. ZVL may also be given to individuals aged >60 years with chronic medical conditions unless they have a severe immunodeficiency; ^3^ ZVL may also be given to individuals aged >60 years with a history of HZ infection to prevent recurrence; ^4^ These guidelines were published after the ZEN workshop was held in August, 2023; ^5^ RZV not recommended for pregnant individuals; ^6^ ZVL contraindicated in patients with immunocompromising conditions and/or on immunosuppressive therapies. ART: antiretroviral therapy; HIV: human immunodeficiency virus; HZ: herpes zoster; ZEN: Zoster Experts’ Network; ZVL: zoster vaccine live.

**Table 3:** Barriers to HZ vaccination reported by experts of the ZEN.

Factors Related to HZ Vaccination	Key Barriers
Vaccine awareness	Limited insight on where information is available on HZ vaccines
Clinic discovery	Lack of awareness of the disease and sites to receive vaccination Limited initiatives or proactive strategies addressing the need for vaccination in practice
Registration	No dedicated person/parties Processes unclear at hospitals and clinics
Consultation/prescription	Low awareness of the disease burden and benefits of HZ vaccination among healthcare workers Healthcare workers are unaware of the referral processes for patients to be vaccinated Patients perceive a low disease severity Limited time at patient consultations to discuss vaccination
Patient decision	Low willingness to receive HZ vaccination at the current price point
Administration	Untrained healthcare workers administering HZ vaccines Limited vaccination infrastructure Complicated patient pathway to receive vaccination
Vaccine availability	Lack of alternate funding or installment programs for HZ vaccines
Adherence	Limited data on second dose compliance Limited resources to effectively remind patients and monitor compliance

HZ: herpes zoster.

**Table 4:** Key consensus actions to aid understanding of and reduce the burden of HZ in Southeast Asia.

**‣**	Drive data collection on HZ clinical presentation, epidemiology, and outcomes from public health institutions to inform evidence-based policies across Southeast Asia
**‣**	Support the curation and distribution of medical education programs for healthcare professionals on HZ disease burden and vaccination guidelines
**‣**	Raise awareness in the general public on vaccination as a preventative measure against HZ, particularly for older adults and immunocompromised populations
**‣**	Standardize current practices in the HZ patient pathway, including when HZ vaccination should be recommended
**‣**	Address issues in vaccine availability and affordability in Southeast Asia

HZ: herpes zoster.

## Data Availability

Most of the cited peer-reviewed publications of this study are available via PubMed, and the gray literature was derived from resources available in the public domain.

## Supplementary Materials

The following supporting information can be downloaded at: https://www.mdpi.com/article/10.3390/vaccines12121433/s1, Table S1: Participants of the ZEN scientific workshop (August, 2023); Table S2: Prevalence of HZ at the National Hospital of Dermatology and Venereology, Vietnam (2022–2023); Figure S1: Patient pathway for patients with HZ in Indonesia; Figure S2: Patient pathway for patients with HZ in Malaysia; Figure S3: Patient pathway for patients with HZ in the Philippines; Figure S4: Patient pathway for patients with HZ in Thailand; Figure S5: Patient pathway for patients with HZ in Vietnam. Reference [63] has been cited in supplementary materials, along with reference [7], which is also cited in the main text.

## References

[1] Matthews S., Curran D., Sabater Cabrera E., Boutry C., Lecrenier N., Cunningham A.L., Schmader K. (2023). An Analysis of How Herpes Zoster Pain Affects Health-related Quality of Life of Placebo Patients From 3 Randomized Phase III Studies. Clin. J. Pain.

[2] Lim D.Z.J., Tey H.L., Salada B.M.A., Oon J.E.L., Seah E.-J.D., Chandran N.S., Pan J.Y. (2024). Herpes Zoster and Post-Herpetic Neuralgia-Diagnosis, Treatment, and Vaccination Strategies. Pathogens.

[3] Patil A., Goldust M., Wollina U. (2022). Herpes zoster: A Review of Clinical Manifestations and Management. Viruses.

[4] Brisson M., Edmunds W.J., Law B., Gay N.J., Walld R., Brownell M., Roos L.L., De Serres G. (2001). Epidemiology of varicella zoster virus infection in Canada and the United Kingdom. Epidemiol. Infect..

[5] Insinga R.P., Itzler R.F., Pellissier J.M., Saddier P., Nikas A.A. (2005). The incidence of herpes zoster in a United States administrative database. J. Gen. Intern. Med..

[6] Huang J., Wu Y., Wang M., Jiang J., Zhu Y., Kumar R., Lin S. (2022). The global disease burden of varicella-zoster virus infection from 1990 to 2019. J. Med. Virol..

[7] San Martin P., Aunhachoke K., Batac M.C.F., Lodrono-Lim K., Kwanthitinan C., Santoso D., Fonseka T., Nguyen M., Guzman-Holst A. (2023). Systematic Literature Review of Herpes Zoster Disease Burden in Southeast Asia. Infect. Dis. Ther..

[8] van Oorschot D., Vroling H., Bunge E., Diaz-Decaro J., Curran D., Yawn B. (2021). A systematic literature review of herpes zoster incidence worldwide. Hum. Vaccin. Immunother..

[9] Chen L.K., Arai H., Chen L.Y., Chou M.Y., Djauzi S., Dong B., Kojima T., Kwon K.T., Leong H.N., Leung E.M. (2017). Looking back to move forward: A twenty-year audit of herpes zoster in Asia-Pacific. BMC Infect. Dis..

[10] Centers for Disease Control and Prevention Complications of Shingles. https://www.cdc.gov/shingles/signs-symptoms.

[11] Muñoz-Quiles C., López-Lacort M., Díez-Domingo J., Orrico-Sánchez A. (2020). Herpes zoster risk and burden of disease in immunocompromised populations: A population-based study using health system integrated databases, 2009–2014. BMC Infect. Dis..

[12] Tseng H.F., Bruxvoort K., Ackerson B., Luo Y., Tanenbaum H., Tian Y., Zheng C., Cheung B., Patterson B.J., Van Oorschot D. (2020). The Epidemiology of Herpes Zoster in Immunocompetent, Unvaccinated Adults ≥50 Years Old: Incidence, Complications, Hospitalization, Mortality, and Recurrence. J. Infect. Dis..

[13] Kawai K., Gebremeskel B.G., Acosta C.J. (2014). Systematic review of incidence and complications of herpes zoster: Towards a global perspective. BMJ Open.

[14] Harpaz R., Ortega-Sanchez I.R., Seward J.F. (2008). Prevention of herpes zoster: Recommendations of the Advisory Committee on Immunization Practices (ACIP). MMWR Recomm. Rep..

[15] Curran D., Doherty T.M., Lecrenier N., Breuer T. (2023). Healthy ageing: Herpes zoster infection and the role of zoster vaccination. npj Vaccines.

[16] Buchan S.A., Daneman N., Wang J., Garber G., Wormsbecker A.E., Wilson S.E., Deeks S.L. (2020). Incidence of Hospitalizations and Emergency Department Visits for Herpes Zoster in Immunocompromised and Immunocompetent Adults in Ontario, Canada, 2002–2016. Clin. Infect. Dis..

[17] Kawai K., Yawn B.P. (2017). Risk Factors for Herpes Zoster: A Systematic Review and Meta-analysis. Mayo Clin. Proc..

[18] Marra F., Parhar K., Huang B., Vadlamudi N. (2020). Risk Factors for Herpes Zoster Infection: A Meta-Analysis. Open Forum Infect. Dis..

[19] Steinmann M., Lampe D., Grosser J., Schmidt J., Hohoff M.L., Fischer A., Greiner W. (2024). Risk factors for herpes zoster infections: A systematic review and meta-analysis unveiling common trends and heterogeneity patterns. Infection.

[20] World Health Organization Ageing and Health in the South-East Asia Region. https://www.who.int/southeastasia/health-topics/ageing#:~:text=The%20population%20in%20WHO%20South-East%20Asia%20Region%20is,and%2020.3%25%20by%202030%20and%20by%202050%2C%20respectively.

[21] Pan C.X., Lee M.S., Nambudiri V.E. (2022). Global herpes zoster incidence, burden of disease, and vaccine availability: A narrative review. Ther. Adv. Vaccines Immunother..

[22] Aunhachoke K., Bussaratid V., Chirachanakul P., Chua-Intra B., Dhitavat J., Jaisathaporn K., Kaewkungwal J., Kampirapap K., Khuhaprema T., Pairayayutakul K. (2011). Measuring herpes zoster, zoster-associated pain, post-herpetic neuralgia-associated loss of quality of life, and healthcare utilization and costs in Thailand. Int. J. Dermatol..

[23] Dilly J., Kapantow M., Suling P. (2016). Profil herpes zoster di poliklinik kulit dan kelamin RSUP Prof. Dr. RD Kandou Manado periode Januari–Desember 2013. E-Clinic.

[24] Jara M.A., Roa F.C. (2010). The clinical presentation of herpes zoster patients predictors of postherpetic neuralgia: A year retrospective study at the section of dermatology, University of the Philippines—Philippine General Hospital. J. Philipp. Dermatol. Soc..

[25] Soong S.L., Eow G.B., Suhaimi F., Thankamony S., Neoh C.A. (2021). Post Herpetic Neuralgia—A study of demographics in Cosmopolitan Tertiary Centre in Penang, Malaysia. J. Neurol. Sci..

[26] Kong C.L., Thompson R.R., Porco T.C., Kim E., Acharya N.R. (2020). Incidence Rate of Herpes Zoster Ophthalmicus: A Retrospective Cohort Study from 1994 through 2018. Ophthalmology.

[27] Anderson T.C., Masters N.B., Guo A., Shepersky L., Leidner A.J., Lee G.M., Kotton C.N., Dooling K.L. (2022). Use of Recombinant Zoster Vaccine in Immunocompromised Adults Aged ≥19 Years: Recommendations of the Advisory Committee on Immunization Practices—United States, 2022. Morb. Mortal Wkly. Rep..

[28] Gangcuangco L.M.A., Eustaquio P.C. (2023). The State of the HIV Epidemic in the Philippines: Progress and Challenges in 2023. Trop. Med. Infect. Dis..

[29] Ku H.C., Tsai Y.T., Konara-Mudiyanselage S.P., Wu Y.L., Yu T., Ko N.Y. (2021). Incidence of Herpes Zoster in HIV-Infected Patients Undergoing Antiretroviral Therapy: A Systematic Review and Meta-analysis. J. Clin. Med..

[30] Lai S.W., Liu C.S., Kuo Y.H., Lin C.L., Hwang B.F., Liao K.F. (2021). The incidence of herpes zoster in patients with diabetes mellitus: A meta-analysis of cohort studies. Medicine.

[31] Sun H., Saeedi P., Karuranga S., Pinkepank M., Ogurtsova K., Duncan B.B., Stein C., Basit A., Chan J.C.N., Mbanya J.C. (2022). IDF Diabetes Atlas: Global, regional and country-level diabetes prevalence estimates for 2021 and projections for 2045. Diabetes Res. Clin. Pract..

[32] Chuanchaiyakul N., Thongtang N., Rattanaumpawan P. (2022). Cumulative incidence of and risk factors for herpes zoster among patients with diabetes mellitus: Results from a 10-year nested case-control study. J. Diabetes Complicat..

[33] Varghese L., Standaert B., Olivieri A., Curran D. (2017). The temporal impact of aging on the burden of herpes zoster. BMC Geriatr..

[34] Massengill J.S., Kittredge J.L. (2014). Practical considerations in the pharmacological treatment of postherpetic neuralgia for the primary care provider. J. Pain Res..

[35] Yeoh C., Chan L., Tan W., Wee H. (2016). Clinical Presentation and Outcome of Herpes Zoster Infection in a Tertiary Dermatology Outpatient Referral Clinic in Malaysia. Malays. J. Dermatol..

[36] Fan H.R., Zhang E.M., Fei Y., Huang B., Yao M. (2023). Early Diagnosis of Herpes Zoster Neuralgia: A Narrative Review. Pain Ther..

[37] Boutry C., Hastie A., Diez-Domingo J., Tinoco J.C., Yu C.J., Andrews C., Beytout J., Caso C., Cheng H.S., Cheong H.J. (2022). The Adjuvanted Recombinant Zoster Vaccine Confers Long-Term Protection Against Herpes Zoster: Interim Results of an Extension Study of the Pivotal Phase 3 Clinical Trials ZOE-50 and ZOE-70. Clin. Infect. Dis..

[38] Strezova A., Diez-Domingo J., Al Shawafi K., Tinoco J.C., Shi M., Pirrotta P., Mwakingwe-Omari A. (2022). Long-term Protection Against Herpes Zoster by the Adjuvanted Recombinant Zoster Vaccine: Interim Efficacy, Immunogenicity, and Safety Results up to 10 Years After Initial Vaccination. Open Forum Infect. Dis..

[39] Centers for Disease Control and Prevention Shingrix Recommendations. https://www.cdc.gov/shingles/hcp/vaccine-considerations.

[40] Oxman M.N., Levin M.J., Johnson G.R., Schmader K.E., Straus S.E., Gelb L.D., Arbeit R.D., Simberkoff M.S., Gershon A.A., Davis L.E. (2005). A vaccine to prevent herpes zoster and postherpetic neuralgia in older adults. N. Engl. J. Med..

[41] Mwakingwe-Omari A., Lecrenier N., Naficy A., Curran D., Posiuniene I. (2023). Recombinant zoster vaccine in immunocompetent and immunocompromised adults: A review of clinical studies. Hum. Vaccin. Immunother..

[42] Dagnew A.F., Ilhan O., Lee W., Woszczyk D., Kwak J.Y., Bowcock S., Sohn S., Rodriguez Macías G., Chiou T.J., Quiel D. (2019). Immunogenicity and safety of the adjuvanted recombinant zoster vaccine in adults with haematological malignancies: A phase 3, randomised, clinical trial and post-hoc efficacy analysis. Lancet Infect. Dis..

[43] Bastidas A., de la Serna J., El Idrissi M., Oostvogels L., Quittet P., López-Jiménez J., Vural F., Pohlreich D., Zuckerman T., Issa N.C. (2019). Effect of recombinant zoster vaccine on incidence of herpes zoster after autologous stem cell transplantation: A randomized clinical trial. JAMA.

[44] Warren-Gash C., Breuer J. (2019). Herpes zoster in people who are immunocompromised: What are the options for prevention?. Lancet Infect. Dis..

[45] Indonesia Society of Internal Medicine (2023). Adult Immunization Schedule—Recommendations from the Adult Immunization Task Force (PAPDI). https://satgasimunisasipapdi.com/jadwal-imunisasi-dewasa.

[46] National Health Service Shingles Vaccine. https://www.nhs.uk/conditions/vaccinations/shingles-vaccination.

[47] Malaysian Society of Infectious Diseases & Chemotherapy (2020). Guidelines for Adult Immunization.

[48] Malaysian Society of Geriatric Medicine (2024). Position Statement—The Herpes Zoster Vaccine. https://www.msgm.com.my/herpes-zoster-vaccine.

[49] Ministry of Health Malaysia (2014). Immunizaton Schedule for the Elderly. http://www.myhealth.gov.my/en/immunization-schedule-for-the-elderly.

[50] Ministry of Health Malaysia (2023). Handbook of Kidney Replacement.

[51] Philippine Society of Microbiology and Infectious Disease (2018). Philippine Clinical Practice Guidelines for Adult Immunization. https://www.psmid.org/clinical-practice-guidelines-for-adult-immunization-2018.

[52] Periodic Health Examination Task Force (2023). Philippine Guidelines on Periodic Health Examination—Immunization for Adults. https://phex.ph.

[53] Infectious Disease Association of Thailand (IDAT) (2023). Recommended Adult and Elderly Immunization Schedule. https://www.idthai.org/Contents/Views/?d=ZTue!17!4!!390!BiuJHPad.

[54] Vietnam Ministry of Health (2023). Guideline on Diagnosis and Treatment of Dermatological Disease. https://luatvietnam.vn/y-te/quyet-dinh-4416-qd-byt-2023-huong-dan-chan-doan-va-dieu-tri-cac-benh-da-lieu-281365-d1.html.

[55] Badur S., Senol E., Azap A., Yesiloglu C., Ozakay A., Ozturk S., Guzman-Holst A. (2023). Herpes Zoster Burden of Disease and Clinical Management in Turkey: A Comprehensive Literature Review. Infect. Dis. Ther..

[56] Yawn B.P., Loskutova N.Y., Merrill D.D., Martinez S., Callen E., Cotton J., Carroll J.K., Williams D. (2022). Health Care Professionals’ Herpes Zoster Awareness and Vaccine Recommendations for Patients with COPD. Chronic Obstr. Pulm. Dis..

[57] Wilson S.L., Wiysonge C. (2020). Social media and vaccine hesitancy. BMJ Glob. Health.

[58] Seraj Ahmad F.N., Khor S.K., Lim J., Roth S., Dewi S., Becerra F. (2022). Southeast Asia needs a revolving fund for vaccines. Lancet Glob. Health.

[59] Chayangsu O., Jiamton S., Leeyaphan C., Prasertworonun N., Omcharoen V., Kulthanan K. (2016). Willingness to pay, quality of life, and knowledge on herpes zoster among Thai patients prior Zoster vaccine era. Southeast Asian J. Trop. Med. Public Health.

[60] Correa R.A., Arancibia F., De Ávila Kfouri R., Chebabo A., García G., Gutiérrez Robledo L.M., Lopardo G., Nemerovsky J., Pérez C.M., Rendon A. (2024). Understanding the Burden of Respiratory Syncytial Virus in Older Adults in Latin America: An Expert Perspective on Knowledge Gaps. Pulm. Ther..

[61] Badur S., Ozudogru O., Khalaf M., Ozturk S., Albreiki S., Al Awaidy S., Guzman-Holst A. (2023). Epidemiology of Varicella Zoster Virus and Herpes Zoster Virus in Gulf Cooperation Council Countries: A Review of the Literature. Infect. Dis. Ther..

[62] Fei H., Ruoyu L. (2022). Call for attention to the standardized management of herpes zoster. Natl. Med. J. China.

[63] Tangcharoensathien V., Sachdev S., Viriyathorn S., Sriprasert K., Kongkam L., Srichomphu K., Patcharanarumol W. (2022). Universal access to comprehensive COVID-19 services for everyone in Thailand. BMJ Glob. Health.

