# Supplementary material for: The Herpes Zoster Patient Pathway and Gaps in Current Vaccination Guidelines in Southeast Asia: Summary of a Zoster Experts’ Network Scientific Workshop
Source: Vaccines (Basel). 2024 Dec 19;12(12):1433. doi: 10.3390/vaccines12121433 (PMC11728494; doi:10.3390/vaccines12121433)
Supplement: Supplementary file 1 [file vaccines-12-01433-s001.zip › vaccines-3289647-supplementary.pdf]

SUPPLEMENTARY MATERIALS

Table S1. Participants of the ZEN scientific workshop (August, 2023)

Participant	Country	Specialty/Role
Dr. Gyneth Lourdes Bibera	Emerging Markets region (GSK employee)	Medical Director, Regional Vaccines, Emerging Markets
Dr. Desiree Van Oorschot	Global (GSK employee)	Senior Director, Value evidence and outcomes, Emerging Markets
Dr. Peter San Martin	Global (GSK employee)	Manager, Value Evidence and outcomes, Emerging Markets
Dr. Bella Dragova-Maurin	Global (GSK employee)	Associate Director, Publications Lead
Dr. Deliana Permatasari	Indonesia (GSK employee)	Medical Director, Vaccines
Dr. Dicky Teguh Santoso	Indonesia (GSK employee)	Senior Medical Affairs Manager
Dr. Thanabalan Fonseka	Malaysia (GSK employee)	Medical Director, Vaccines
Dr. Tan Li Ling	Malaysia (GSK employee)	Senior Medical Affairs Manager
Dr. Joselito Javier	Philippines (GSK employee)	Senior Medical Affairs Manager
Dr. Bussakorn Mahakkanukrauh	Thailand (GSK employee)	Country Medical Director
Dr. Phatu Boonmahittisut	Thailand (GSK employee)	Vaccines Medical Head
Dr. Waraporn Boonpramook	Thailand (GSK employee)	Medical Advisor
Roongaroon Phuangtham	Thailand (GSK employee)	Medical Scientific Liaison
Dr. Elena DeAngelis	Vietnam (GSK employee)	Country Medical Director
Dr. Thanh Tuyen Dang Thi	Vietnam (GSK employee)	Medical Affairs Manager Lead
Dr. Pham Anh Duc	Vietnam (GSK employee)	Medical Affairs Manager
Prof. Dr. Iris Rengganis	Indonesia	(Professor) Internist Allergy & Immunology Consultant
Prof. Dr. Lili Legiawati, Sp.DVE, Subsp.D.K.E., FINSDV, FAADV	Indonesia	(Professor) Dermatologist Venereologist and Aesthetics Consultant of Cosmetics and Aesthetics
Prof. Dr. Afif Nurul Hidayati, Sp.DVE, Subsp.Ven, FINSDV, FAADV	Indonesia	(Professor) Dermatologist Venereologist and Aesthetics, Consultant of Venereology
Dr. Isti Suharjanti	Indonesia	Neurologist Consultant
Dr. Nurwestu Rusetiyanti, Sp.DVE, Subsp.Ven, FINSDV, FAADV	Indonesia	Dermatologist Venereologist and Aesthetics Consultant of Venereology
Dr. Peter Ch'ng Wee Beng	Malaysia	Dermatology
Prof. Dr. Zamberi bin Sekawi	Malaysia	Virologist
Dr. Rasool Shaik bin Sehu Mohamed	Malaysia	Aesthetic Physician
Prof. Dr. Chin Ai-Vyrn	Malaysia	Geriatrics
Prof. Dr. Sasheela A/P Sri La Sri Ponnampalavanar	Malaysia	Infectious Disease
Dr. Mario Panaligan	Philippines	Infectious Disease
Dr. Maria Christina Filomena Batac	Philippines	Dermatology
Dr. Geraldine Zamora-Abrahan	Philippines	Rheumatology
Dr. Edsel Maurice Salvana	Philippines	Infectious Disease
Prof. Dr. Nuntana Kasitanon	Thailand	Rheumatology
Prof. Pinyo Rattanaumpawan	Thailand	Infectious Disease

Participant	Country	Specialty/Role
Assoc. Prof. Piroon Mootsikapun	Thailand	Infectious Disease
Prof. Terapong Tantawichien	Thailand	Infectious Disease
Dr. Suda Sibunruang	Thailand	Immunology
Assoc. Prof. Pham Quang Thai	Vietnam	Epidemiology
Assoc. Prof. Do Duy Cuong	Vietnam	Infectious Disease
Dr. Trinh Minh Trang	Vietnam	Dermatology
Dr. Nguyen Hien Minh	Vietnam	Immunization
Dr. Le Ba Ngoc	Vietnam	Endocrinology

Table S2. Prevalence of HZ at the National Hospital of Dermatology and Venereology, Vietnam (2022–2023[a])

	Year	
	**2022**	**2023[a]**
Prevalence of HZ		
Patients examined, N (%)	398,808 (100.0)	185,429 (100.0)
Patients with HZ, n (%)	3,782 (0.9)	1,729 (0.9)
Q1	523	956
Q2	976	773
Q3	1,197	–
Q4	1,086	–
Characteristics of patients with HZ		
N	3,782 (100)	1,729 (100)
In-patient, n (%)	82 (2.2)	143 (8.3)
Outpatient, n (%)	3,700 (97.8)	1,586 (91.8)
Sex, female, n (%)	2,082 (55.1)	981 (56.7)
Pregnant, n (%)	0 (0.0)	2 (0.1)
Age, n (%)		
<1 year	1 (0.03)	0 (0.0)
<12 years	59 (1.6)	31 (1.8)
>50 years	1,749 (46.2)	1,237 (71.5)
>70 years	862 (22.8)	385 (22.3)
PHN, n (%)	1,582 (41.8)	690 (39.9)
Aged <1 year	1 (0.03)	0 (0.0)
Aged <12 years	2 (0.05)	1 (0.1)
Aged >50 years	1,412 (37.3)	618 (35.7)
Aged >70 years	519 (13.7)	218 (12.6)

[a]Data were available up to June 6, 2023. HZ: herpes zoster; PHN: postherpetic neuralgia; Q: quarter.

Figure S1. Patient pathway for patients with HZ in Indonesia

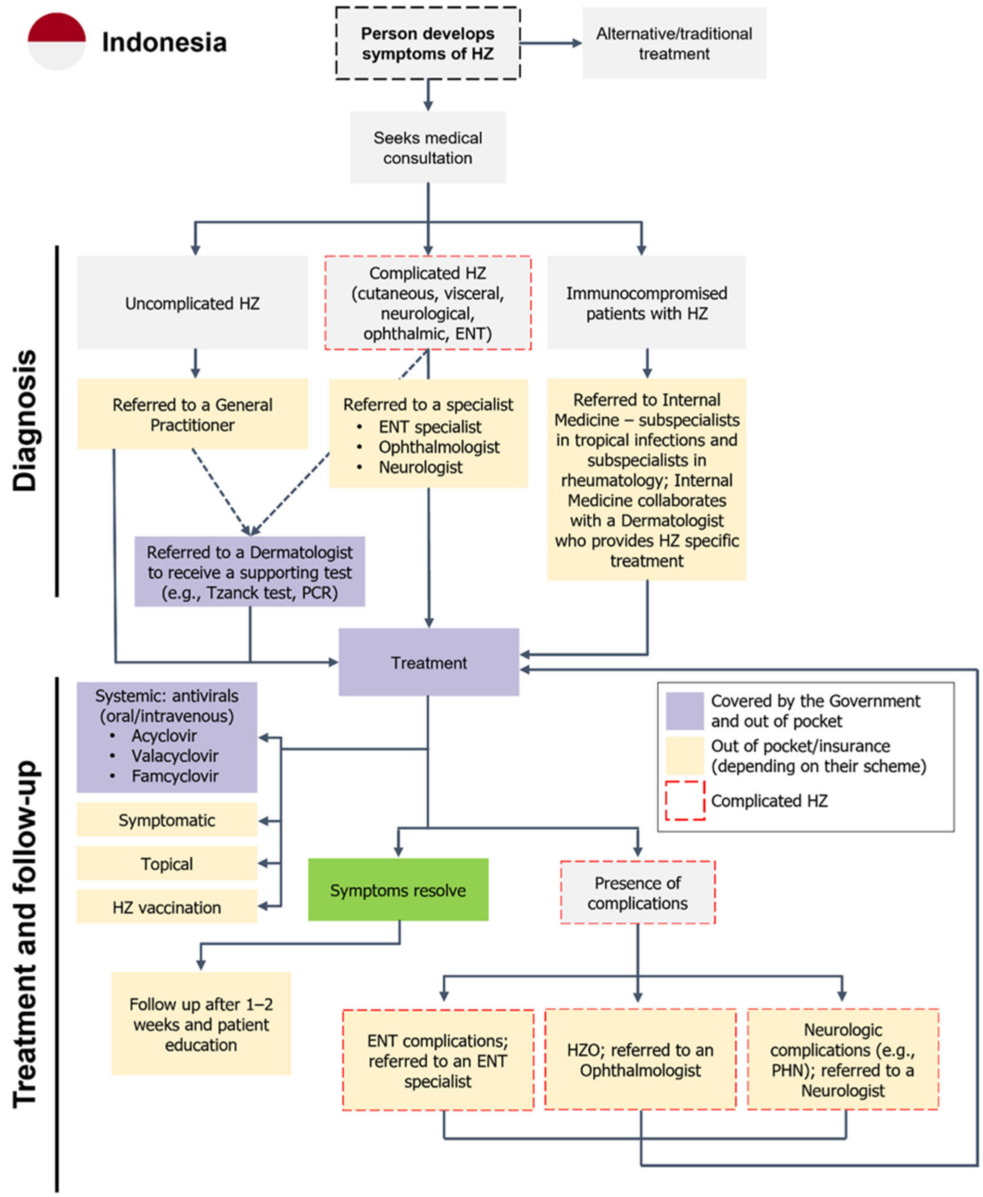

Patient pathway developed based on discussions held at the ZEN scientific workshop, where clinical experts and public health specialists collectively outlined the current patient pathway for individuals with symptoms of HZ based on their own experience in Indonesia. ENT: ears, nose and throat; HZ: herpes zoster; HZO: HZ ophthalmicus; PCR: polymerase chain reaction; PHN: postherpetic neuralgia; ZEN: Zoster Experts' Network.

Figure S2. Patient pathway for patients with HZ in Malaysia

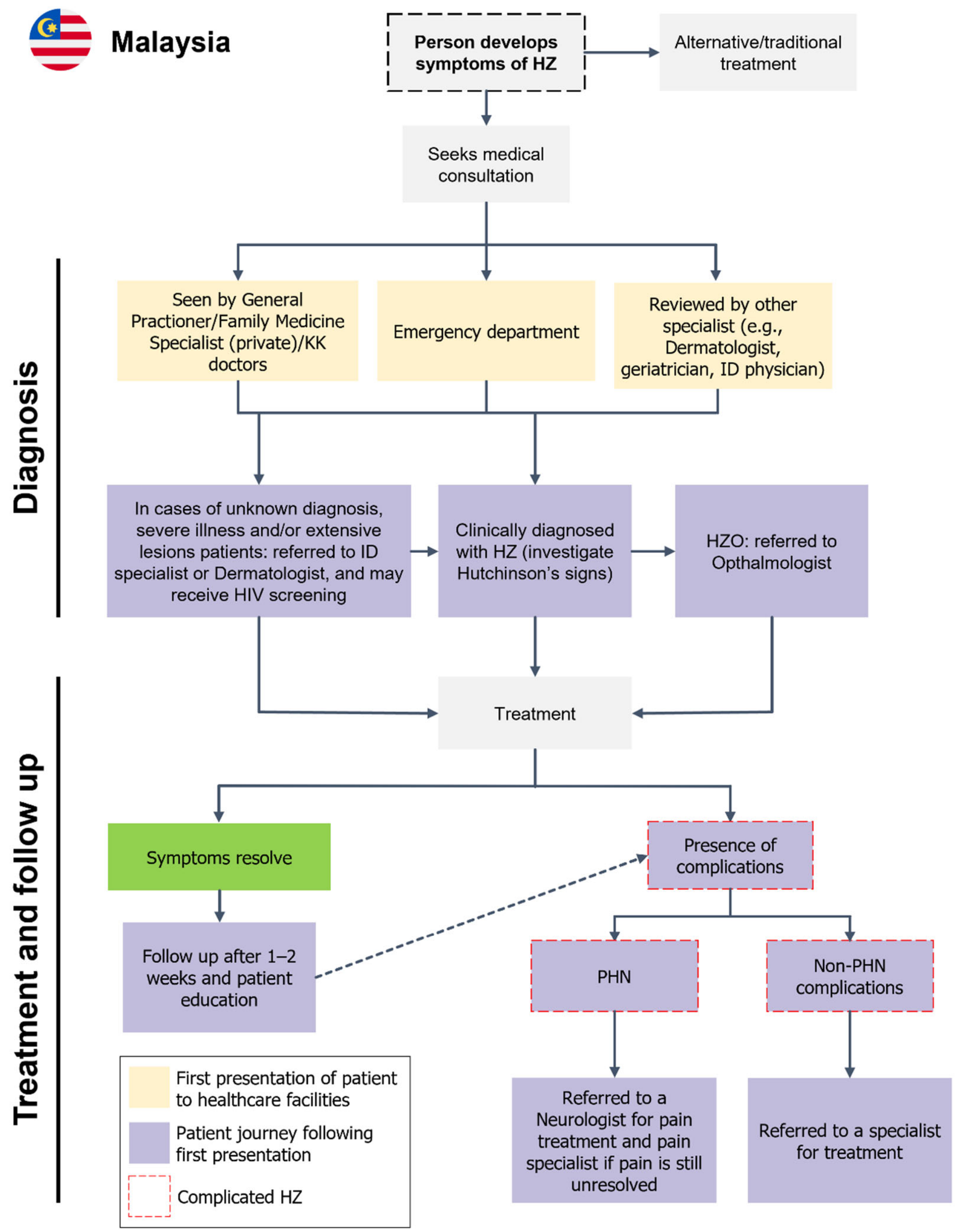

Patient pathway developed based on discussions held at the ZEN scientific workshop, where clinical experts and public health specialists collectively outlined the current patient pathway for individuals with symptoms of HZ based on their own experience in Malaysia. HZ: herpes zoster; HZO: HZ ophthalmicus; ID: infectious disease; KK: Klinik Kesihatan (Governmental primary care); PHN: postherpetic neuralgia; ZEN: Zoster Experts' Network.

Figure S3. Patient pathway for patients with HZ in the Philippines

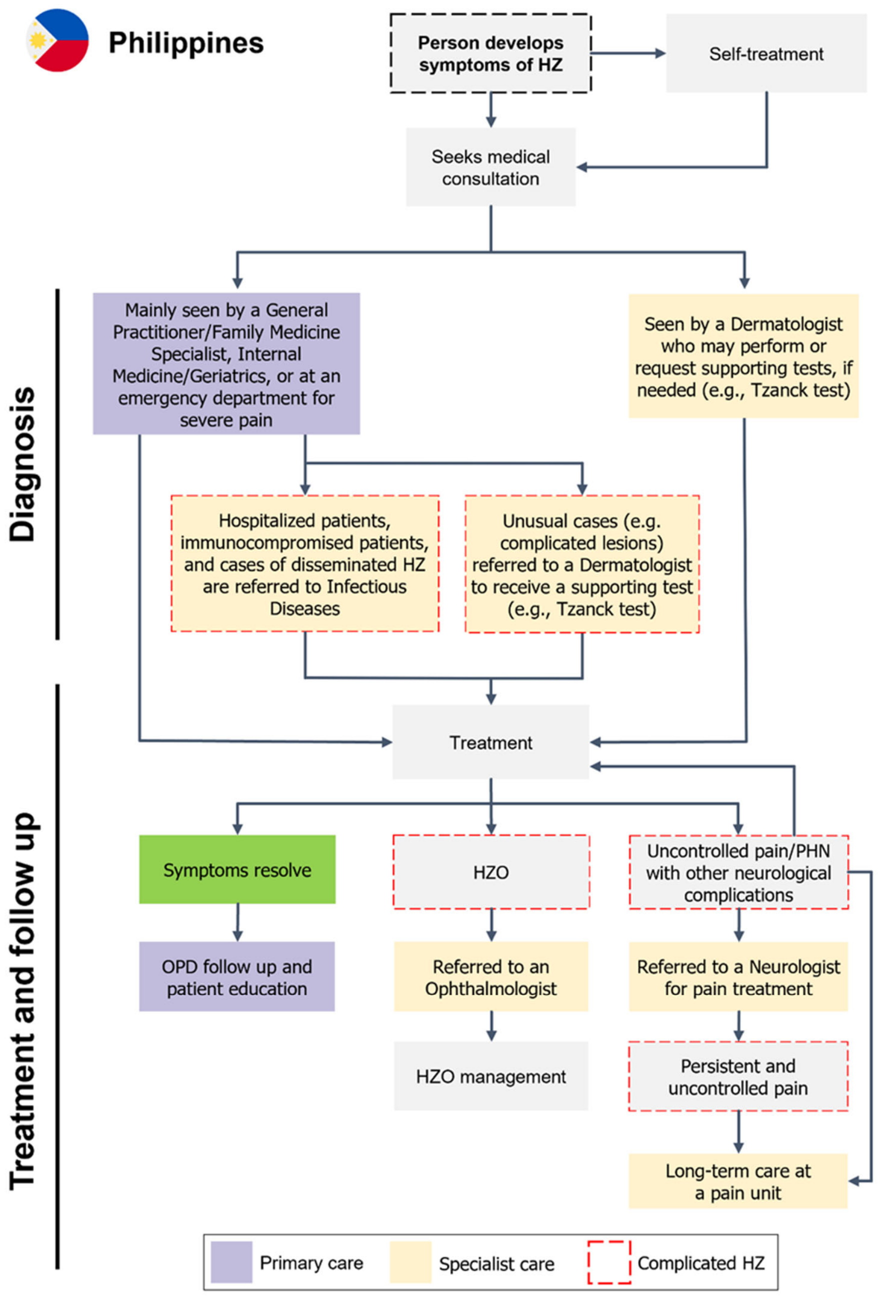

Patient pathway developed based on discussions held at the ZEN scientific workshop, where clinical experts and public health specialists collectively outlined the current patient pathway for individuals with symptoms of HZ based on their own experience in the Philippines. HZ: herpes zoster; HZO: HZ ophthalmicus; OPD: outpatient department; PCR: polymerase chain reaction; PHN: postherpetic neuralgia; ZEN: Zoster Experts' Network.

Figure S4. Patient pathway for patients with HZ in Thailand

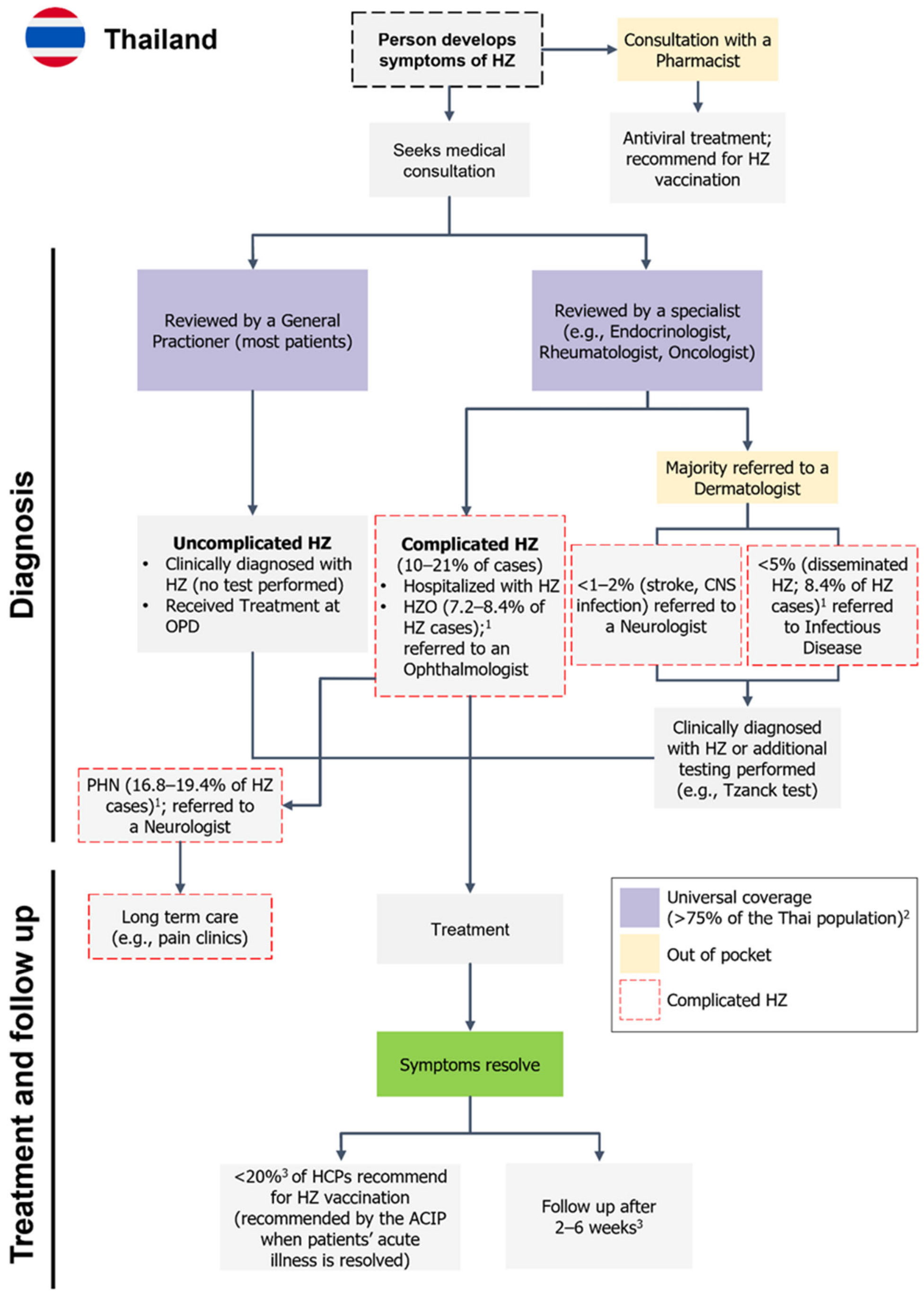

Patient pathway developed based on discussions held at the ZEN scientific workshop, where clinical experts and public health specialists collectively outlined the current patient pathway for individuals with symptoms of HZ based on their own experience in Thailand. [1]Data reported from San Martin et al. (2023);[1] [2]Data reported from Tangcharoensathien et al. (2022);[2] [3]Data reported from informal interviews with five Thai clinicians. ACIP: Advisory Committee on Immunization Practices; CNS: central nervous system; HCP: healthcare professional; HZ: herpes zoster; HZO: HZ ophthalmicus; ID: infectious disease; OPD: outpatient department; PHN: postherpetic neuralgia; ZEN: Zoster Experts' Network.

Figure S5. Patient pathway for patients with HZ in Vietnam

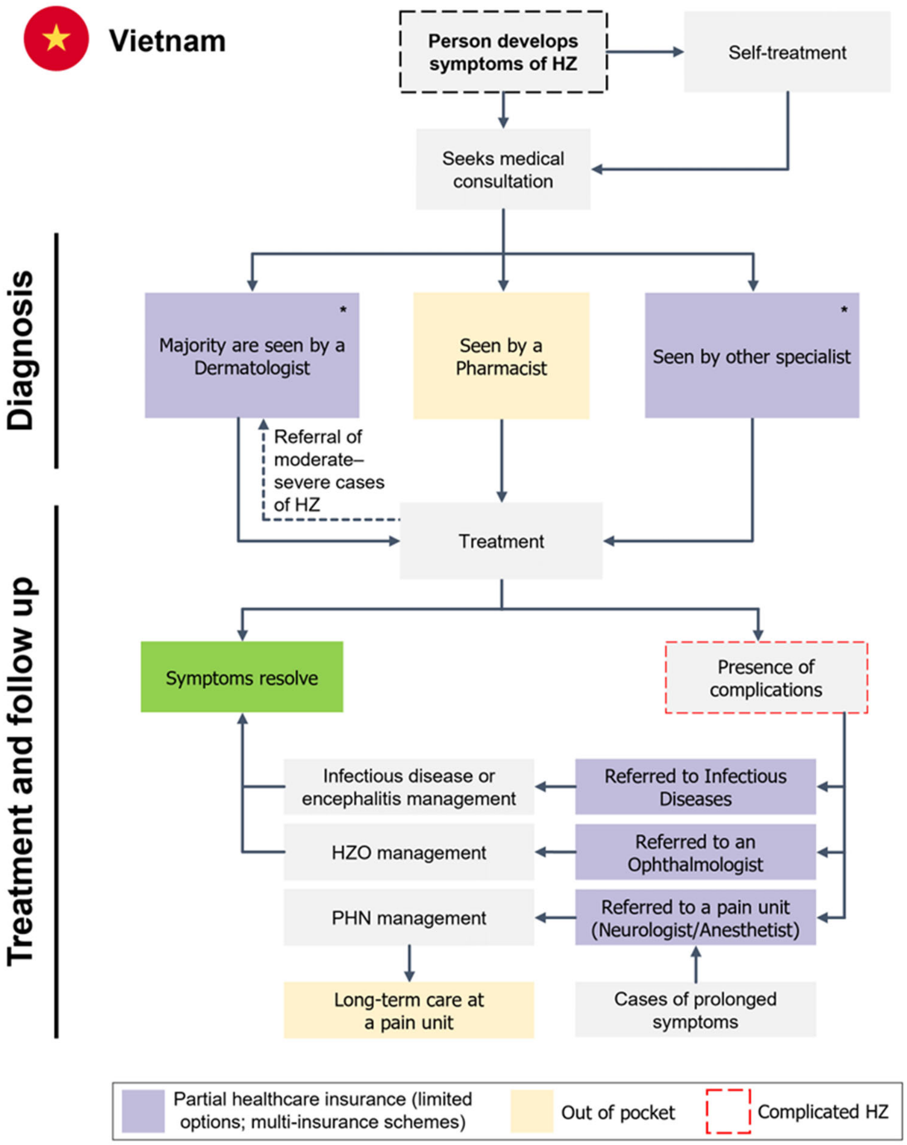

Patient pathway developed based on discussions held at the ZEN scientific workshop, where clinical experts and public health specialists collectively outlined the current patient pathway for individuals with symptoms of HZ based on their own experience in Vietnam. *From the experience of the health professionals at the ZEN workshop, many patients seek diagnosis and treatment out of pocket due to inconvenient procedures associated with insurance schemes. HZ: herpes zoster; HZO: HZ ophthalmicus; PHN: postherpetic neuralgia; ZEN: Zoster Experts' Network.

REFERENCES

1. San Martin, P.; Aunhachoke, K.; Batac, M.C.F.; Lodrono-Lim, K.; Kwanthitinan, C.; Santoso, D.; Fonseka, T.; Nguyen, M.; Guzman-Holst, A. Systematic Literature Review of Herpes Zoster Disease Burden in Southeast Asia. *Infect Dis Ther* **2023**, *12*, 1553-1578, doi:10.1007/s40121-023-00822-0.
2. Tangcharoensathien, V.; Sachdev, S.; Viriyathorn, S.; Sriprasert, K.; Kongkam, L.; Srichomphu, K.; Patcharanarumol, W. Universal access to comprehensive COVID-19 services for everyone in Thailand. *BMJ Glob Health* **2022**, *7*, doi:10.1136/bmjgh-2022-009281.
